# Neonatal Thyroid-Stimulating Hormone Reference Intervals in Multi-Ethnics Population

**DOI:** 10.3390/children12010104

**Published:** 2025-01-17

**Authors:** Hery Priyanto, Fauqa Arinil Aulia, Hartono Kahar, Muhammad Faizi, Ferdy Royland Marpaung, Aryati Aryati

**Affiliations:** 1 Clinical Pathology Sub-Specialization Program, Department of Clinical Pathology Dr. Soetomo Academic Hospital, Faculty of Medicine Universitas Airlangga, Surabaya 60132, Indonesia; hery.priyanto-2022@fk.unair.ac.id; 2 Department of Clinical Pathology Dr. Soetomo Academic Hospital, Faculty of Medicine Universitas Airlangga, Surabaya 60132, Indonesia; fauqa.arinil.aulia@gmail.com (F.A.A.); hartono.kahar@fk.unair.ac.id (H.K.); ferdy.royland.marpaung-2022@fk.unair.ac.id (F.R.M.); 3 Department of Child Health, Dr. Soetomo Academic Hospital, Faculty of Medicine Universitas Airlangga, Surabaya 60132, Indonesia; muhammad.faizi@fk.unair.ac.id; 4 Doctoral Program of Medical Science, Faculty of Medicine, Universitas Airlangga, Surabaya 60132, Indonesia

**Keywords:** reference intervals, thyroid stimulating hormone, dry blood spot, child health

## Abstract

(1) Background: This study is designed to establish thyroid-stimulating hormone (TSH) reference intervals tailored to different neonatal age groups and Indonesian local populations. (2) Methods: Dried blood spot neonatal TSH values, from 1 January 2022 to 31 December 2023, were used to establish the neonatal TSH reference intervals partitioned by sex, gestational age, and ethnic group at different neonatal ages. (3) Results: A significant difference in the reference intervals value was observed in sex, gestational ages, and parental ethnicity groups in different neonatal age subgroups (*p* < 0.05). Male reference intervals were significantly higher than those of females at all neonatal ages. Late and post-term gestational age categories reference intervals were higher than early and full-term. Among the ethnic groups, Madurese had a higher upper limit TSH reference interval. (4) Conclusions: Our neonatal TSH reference intervals were needed to provide a reference adapted to the local population of Indonesia.

## 1. Introduction

Thyroid hormones are essential for normal brain growth and development, especially during the first year of life. Hypothyroidism in this critical period is a significant and preventable cause of intellectual impairment globally [1,2]. Congenital hypothyroidism is a disorder characterized by an insufficient amount of thyroid hormones that are present at birth, resulting in inadequate levels for the proper growth and development of body tissues [3]. Postponed treatment results in impaired skeletal and neural growth, which causes dwarfism and chronic brain damage or cretinism [4].

Hypothyroidism is typically characterized by minimal or mild symptoms at birth, often appearing asymptomatic in clinical settings [5]. Therefore, it is imperative to perform screenings for neonatal hypothyroidism to ensure timely diagnosis and treatment of the condition [6]. Neonatal screening programs have been implemented in various developed countries since the 1970s. There were 595 cases of congenital hypothyroidism in Indonesia in 2010. Most of these cases were diagnosed later, leading to delayed motor development, cognitive growth, and intellectual deficits. A previous study showed that a total of 1708 infants were detected with elevated screening results, of which 73 cases were diagnosed with congenital hypothyroidism across 11 provinces in Indonesia. The study also showed that the ratio of 1:2736 is equivalent to the global ratio of 1:3000 births [6].

The thyroid-stimulating hormone (TSH) assay is crucial in evaluating thyroid function. Despite its clinical importance, interpreting TSH assay results in pediatric populations is often challenging due to the lack of well-established reference intervals specific to children [7]. The European Thyroid Association guidelines for addressing subclinical hypothyroidism in children suggest utilizing age-specific reference intervals [8]. Measuring reference intervals directly in children is difficult due to the many physiological changes associated with growth and development during childhood and adolescence [9]. To establish specific reference intervals for various laboratory tests in pediatric patients, it is necessary to divide the data based on age and sex appropriately [9]. Recruiting an adequate size and healthy pediatric cohort for stratification by relevant variables is quite difficult due to the substantial costs required [9]. To achieve optimal therapeutic outcomes, it is essential to establish precise reference intervals for TSH specific to different age groups and characteristics of the local population [10]. This study was carried out to determine the TSH reference intervals in newborns following the Clinical Laboratory Standards Institute (CLSI) guidelines, precisely the EP28-A3c standard, as part of the national program for screening congenital hypothyroidism in East Java province, Indonesia.

## 2. Materials and Methods

**Figure 1:**
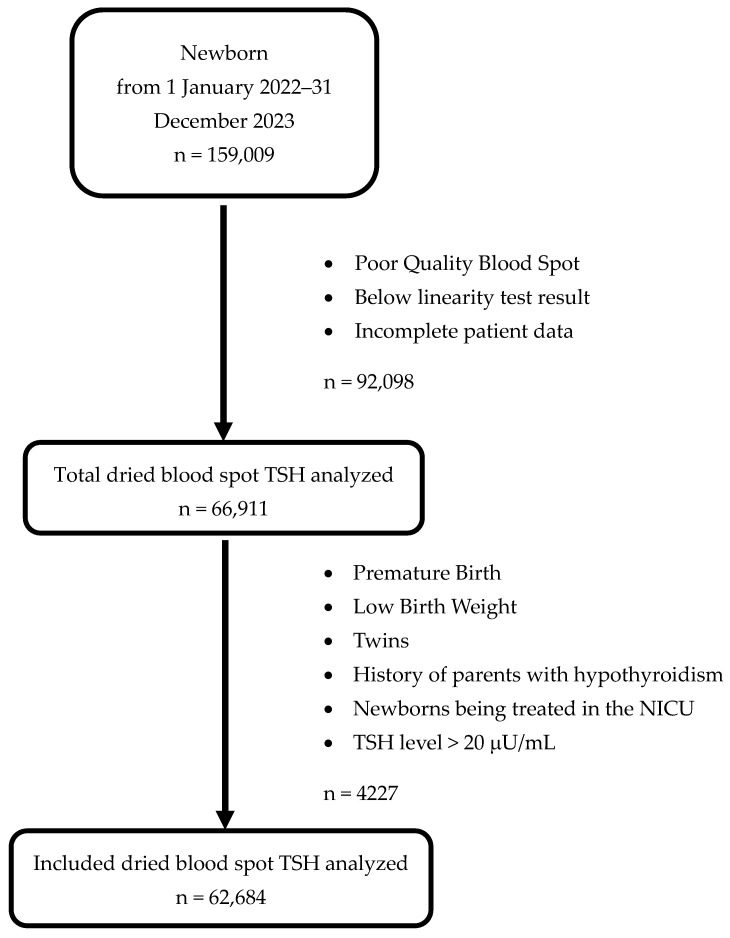
Flowchart dried blood spot TSH included in the study.

This retrospective study used samples from dried blood spots on 903 grade Whatman Filter Paper (Wallay Oy, Turku, Finland) for hypothyroid newborn screening in East Java Province, Indonesia. The TSH examination was performed at the referral laboratory located at Dr. Soetomo Academic General Hospital in Surabaya, East Java, Indonesia. The TSH values of the dried blood spots stored in the hospital’s data center were used to determine the reference intervals for neonatal TSH levels from 1 January 2022 to 31 December 2023. According to the Clinical Laboratory Standards Institute (CLSI) EP28-A3c guideline, at least 120 samples must be examined using non-parametric statistical methods with a 90% confidence interval to determine the reference intervals [11]. This study used 159,009 newborn dry blood spot samples from newborn screening examinations for the TSH examination. The samples that met the criteria for determining reference intervals, namely single birth, normal birth weight, and term gestational age, were 62,684, as illustrated in Figure 1. This study excluded twin births, low birth weights, premature neonates, history of parents with hypothyroidism, TSH level ≥ 20 μU/mL, and those being treated in the Neonatal Intensive Care Unit (NICU). Reference intervals were partitioned based on sex, gestational age, and parental ethnicity in different neonatal ages. Because of physiological variations, including thyroid function after birth, the neonatal ages were divided into subgroups: ≤24 h, >24–48 h, >48–72 h, and >72 h. Describing a standardized method for calculating gestational age is essential to simplify data reporting, ensure the provision of high-quality healthcare, and promote clinical research. To resolve the inconsistency in newborn outcomes between 37 0/7 weeks of gestation and 42 0/7 weeks of gestation, a task force assembled in late 2012 proposed substituting “term” with “early-term” (37 0/7 weeks through 38 6/7 weeks), “full-term” (39 0/7 weeks through 40 6/7 weeks), “late-term” (41 0/7 weeks through 41 6/7 weeks), and “post-term” (42 0/7 weeks and beyond) to provide a more precise description of deliveries that take place at or beyond 37 0/7 weeks of gestation [12]. The origin of the parental ethnicity was divided into Javanese, Madurese, mixex Javanese and Madurese, and others (non-Javanese, non-Madurese).

### 2.1. Biochemical Analysis

The TSH level was measured using the Genetic Screening Processor (GSP) Neonatal hTSH blood spot time-resolved fluoroimmunoassay from PerkinElmer^®^ (PerkinElmer, Wallac Oy, Turku, Finland). The method is linear for TSH concentration from 0.66 μU/mL to 375 μU/mL of blood. TSH-WHO standard material (WHO 3rd IS for TSH 81/565, National Institute for Biological Standards and Control (NIBSC), South Mimms, Hertfordshire, UK) was used to prepare primary calibrators for the GSP Neonatal hTSH kit. The Newborn Screening Quality Assurance Program (NSQAP) from the Centers for Disease Control and Prevention (CDC) indicated acceptable results for the first quarter of 2024. The Internal Quality Control inter-assay coefficients of variation (CV) were between 8.23% and 8.79% during study enrolment.

### 2.2. Statistical Analysis

Statistical calculations used Statistical Package for the Social Sciences (SPSS^®^) software for Windows (version 27) and MedCalc^®^ software for Windows (version 20.218). The Mann–Whitney U and Kruskal–Wallis H tests were used to evaluate the significance of the data distribution of neonatal age concerning sex, gestational age, and parental ethnicity. The Mann–Whitney U test determined the association of two independent variables (sex), and the Kruskall—Wallis test determined the association of three or more independent variables (gestational ages and parental ethnicity). Data TSH results were carried out normality tests using the Kolmogorov–Smirnov test with a significant level of *p* < 0.05. The data were presented as median, and the reference intervals were displayed in percentile values 2.5th (lower limit) and 97.5th (upper limit) with 90% confidence intervals when the data distribution was non-Gaussian. The outlier detection method for determining reference intervals was proposed by Tukey (1977) as recommended by the Clinical Laboratory Standards Institute (CLSI) EP28-A3c guideline [11]. Reference intervals were established based on transformed data when the data did not follow a normal distribution. We used the Box–Cox transformation in conjunction with Tukey’s outlier detection technique. Mann–Whitney U Test was used to compare the median of the two groups of different neonatal ages. The Kruskal–Wallis test compared the median in more than two groups of different neonatal ages. A significant *p*-value was less than 0.05. Quantile regression analysis was used to assess the influence of neonatal age on the distribution of TSH levels. This method was chosen because it is particularly well-suited for estimating specific quantiles (e.g., 2.5th and 97.5th percentiles) and does not assume a normal data distribution. The quantile regression model was applied to calculate the change in TSH percentiles across different neonatal age groups. The regression coefficients were calculated for the 2.5th and 97.5th percentiles to observe changes across age groups. A *p*-value < 0.05 was considered statistically significant.

### 2.3. Institutional Review Board Statement

This study received approval from the Health Research Ethics Committee of Dr. Soetomo Surabaya Academic Hospital (Ref No: 1608/LOE/301.4.2/III/2024). The Ethics Committee waived the need for consent because our study was considered retrospective.

## 3. Results

**Figure 2:**
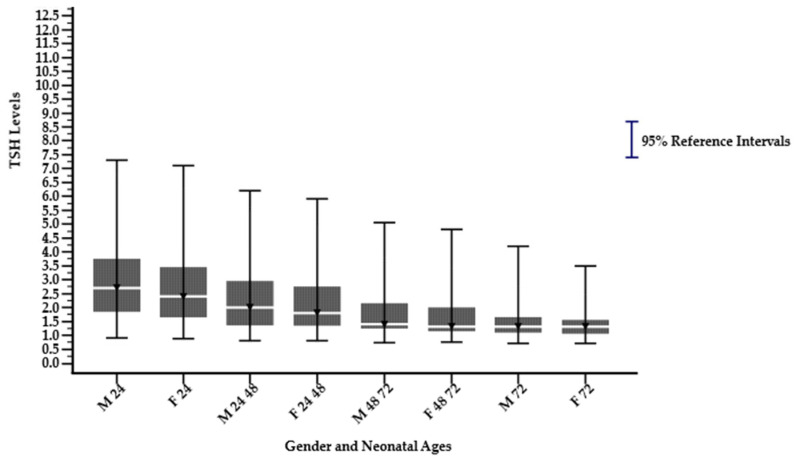
Dried blood spot thyroid-stimulating hormone reference intervals derived from males and females on different neonate age subgroups (hours) (M 24: Males ≤ 24; F 24: Females ≤ 24; M 24 48: Males >24–48; F 24 48: Females >24–48; M 48 72: Males >48–72; F 48 72: Females >48–72; M 72: Males >72; F 72: Females >72).

**Figure 3:**
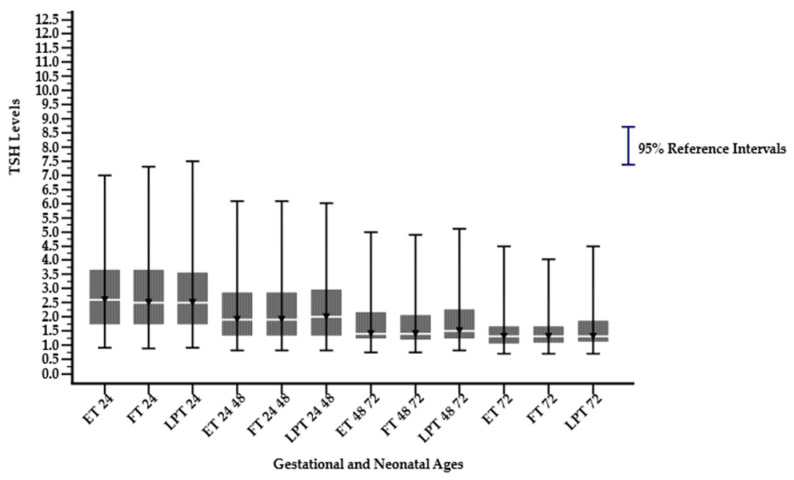
Dried blood spot thyroid-stimulating hormone reference intervals derived from early, full, late, and post-term on different neonate age subgroups (hours). ET 24: Early Term ≤ 24; FT 24: Full Term ≤ 24; LPT 24: Late and Post Term ≤ 24; ET 24 48: Early Term >24–48; FT 24 48: Full Term > 24–48; LPT 24 48: Late and Post Term > 24–48; ET 48 72: Early Term > 48–72; FT 48 72: Full Term > 48–72; LPT 48 72: Late and Post Term > 48–72; ET 72: Early Term > 72; FT 72: Full Term > 72; LPT 72: Late and Post Term > 72.

**Figure 4:**
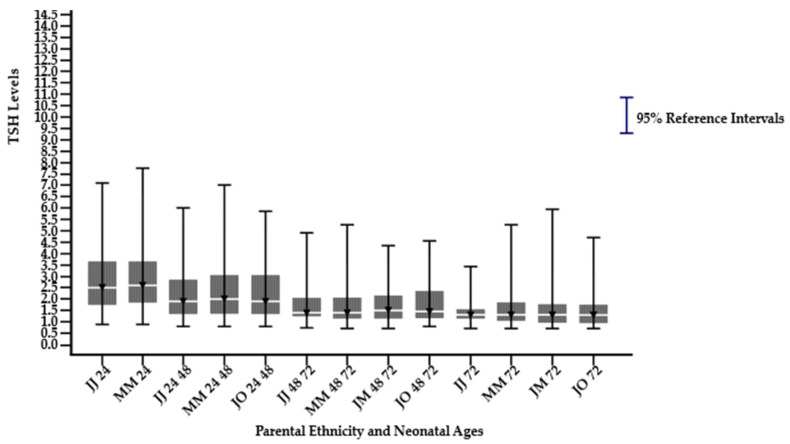
Dried blood spot thyroid-stimulating hormone reference intervals derived from Javanese, Madurese, mixed Javanese–Madurese, and other parental ethnicity on different neonate age subgroups (hours). JJ 24: Javanese and Javanese ≤ 24; MM 24: Madurese and Madurese ≤ 24; JJ 24 48: Javanese and Javanese > 24–48; MM 24 48: Madurese and Madurese > 24–48; JO 24 48: Javanese and others > 24–48; JJ 48 72: Javanese and Javanese > 48–72; MM 48 72: Madurese and Madurese > 48–72; JM 48 72: Javanese and Madurese > 48–72; JO 48 72: Javanese and others > 48–72; JJ 72: Javanese and Javanese > 72; MM 72: Madurese and Madurese > 72; JM 72: Javanese and Madurese > 72; JO 72: Javanese and others > 72.

A significant difference in the median, 2.5th percentile (lower limit), and 97.5th percentile (upper limit) was observed in sex, gestational ages, and parental ethnicity groups in different neonatal age subgroups (*p* < 0.05), as shown in Table 1. The median, 2.5th percentile, and 97.5th percentile TSH levels decreased significantly with increasing neonatal age. The highest median, 2.5th percentile, and 97.5th percentile TSH were observed in neonates ≤ 24 h, followed by a decline across subsequent age groups, namely >24–48, >48–72, and >72 h, except for the parental ethnicity group Javanese-others and Javanese-Madurese, as illustrated in Figure 2, Figure 3 and Figure 4. In the last two groups, not all neonatal ages can be calculated for their reference intervals because they do not meet the minimum requirement of 120 samples, as specified by the Clinical Laboratory Standards Institute (CLSI) EP28-A3c guidelines. Among the ethnic groups, Madurese had a higher 97.5th percentile (upper limit) for the TSH reference interval. The median value was consistently the same at the neonatal age > 72 h for all groups, except for the Javanese–others group.

## 4. Discussion

This study established the newborn TSH reference intervals by analyzing historical hospital laboratory data from the national neonatal screening program in East Java, Indonesia. The data distribution was categorized according to sex, gestational age, neonatal age, and parental ethnicity when collecting dried blood spots. For the sex group, our study found that the male reference intervals were substantially higher than those for females. Our study aligns with prior research [13,14,15,16,17,18]. Nevertheless, multiple investigations had demonstrated no notable disparity in the reference intervals between male and female infants. This could be attributed to the limited sample size and/or the study’s specific emphasis on preterm infants [19,20]. The reasons for the higher levels of newborn TSH in males are still unknown. Nevertheless, this disparity should motivate congenital hypothyroidism screening programs to develop their sex-specific cut-off values [21].

Premature neonates were intentionally excluded from this study due to their vulnerability to hypothyroidism caused by a delayed rise in TSH associated with an immature hypothalamic–pituitary–thyroid axis. The aim was to establish the TSH reference intervals in healthy, full-term neonates. The study revealed significant median, 2.5th, and 97.5th percentile differences in TSH levels across early, full, late, and post-term gestational periods. These results differ from prior investigations in which no substantial disparity was observed [15,19]. The TSH reference intervals for late and post-term gestational age categories were higher than for early and full-term, indicating that the greater the gestational age, the more mature the physiological hormone in the hypothalamic–pituitary–thyroid (HPT) axis [22,23,24,25]. HPT maturation progresses from 35 to 40 weeks of gestation. During this period, there is a noticeable increase in the levels of Thyrotropin Releasing Hormone (TRH) in the hypothalamus, and the progression of the hypothalamic-pituitary portal vascular system reaches maturity [26]. The levels of hypothalamic TRH rise throughout the second trimester. In contrast, the levels of hypothalamic TRH content, pituitary TSH content, and serum TSH gradually increase from the middle of pregnancy to full term. The growth of the thyroid gland increases throughout the third trimester. The volume of the thyroid gland in the human fetus, as determined by ultrasonography in premature babies, is below 0.2 mL before 30 weeks of gestation. However, it increases by 8 to 10 times between 30 and 42 weeks of gestational age [26].

The upper limit of TSH levels was higher in the first 24 h in all groups, except for the Javanese–Madurese and Javanese–other groups. Hence, the reference intervals of TSH at that age were significantly higher than that of neonates after 24 h. These results were consistent with previous studies [27,28,29]. Postnatal TSH levels increase after birth. This surge reaches its highest point, 70 to 100 μU/mL, within the first hour after birth. Over the next 24 to 48 h, the TSH levels gradually decrease. The surge is initiated by severing the umbilical cord and exposing the newborn to a cooler environment outside the womb. [26]. This study’s upper limit reference value was consistently the lowest at the neonatal age > 72 h for all groups, except for the Javanese–Madurese and Javanese–other groups. In the last two groups, it could be caused by smaller subjects compared to other groups, which may lead to greater susceptibility to variation in the data. This result followed the results of the previous study, where the TSH reference intervals were narrower and relatively constant on day 4 [28]. By the end of the first week, the serum TSH concentration is usually below 10.0 μU/mL and falls within the normal range for children and adults by the age of two weeks [28,29].

Indonesia has 1340 ethnic groups spread throughout the Indonesian archipelago. The Javanese are the largest tribe in terms of population. This study participants used newborn neonates in East Java province, the second largest population in Indonesia, with a 14.62 crude birth rate among 1000 East Java residents. It comprises various ethnic groups, with the Javanese ethnic group being the predominant ethnic demographic, whereas the Madurese is the fifth-largest ethnic group [30,31]. According to the total population of East Java from the 2020 Population Census (SP2020), the largest distribution of East Java’s population is found in the Mataraman cultural zone, which is 34.62%, followed by the Arek cultural zone at 30.86%, the Pandalungan cultural zone at 24.67%, and the Madurese cultural zone at 9.85%. Mataraman, Arek, and Pandalungan consist of not only Javanese but also Madurese and other ethnicities [32]. Ethnicity, along with sex and age, is an important factor in determining reference intervals [33]. Among the other ethnic groups, Madurese had a higher upper limit TSH reference. It is due to the higher consumption of goitrogenic food sources (inhibitors of iodine absorption) in the Madurese ethnicity compared to Javanese ethnic groups, in addition to disorders caused by iodine deficiency [34]. Maternal iodine nutrition and thyroid function status can significantly impact fetal and newborns’ TSH levels [35]. Ethnic groups from regions with a historical prevalence of iodine deficiency exhibited the most significant shifts in average TSH concentrations [36]. Prior research had consistently shown that it exhibits a similar pattern, regardless of the specific value used as a cutoff point. Differences in TSH levels among ethnic groups may be attributed to variations in average TSH levels. At the extreme distribution level, such as very high or very low TSH values, the distinctions between ethnic groups become more evident [37].

Due to significant variations in the concentrations of many routinely measured analytes during growth and development, using accurate pediatric reference intervals is essential to avoid misdiagnosis and misclassification of diseases [38]. Establishing accurate reference intervals is inherently important, as optimal reference intervals must be derived from a healthy population and stratified according to key covariates such as age, sex, and ethnicity [38]. Various TSH levels serve as threshold values for congenital hypothyroidism screening worldwide. Several countries have utilized a filter paper TSH cut-off varying from 6 to 17 μU/mL [39,40,41,42,43,44,45,46,47]. If the TSH cut-off points are lowered, this would lead to an increase in the number of newborns being referred, resulting in higher costs and increased anxiety for families. Conversely, if the TSH cut-off points are raised, there is a risk of missing potential positive cases [48]. The Indonesian national congenital hypothyroid screening program used a cut-off value of ≥20 μU/mL for subsequent confirmatory tests with serum FT4 and TSH. In our study, the highest upper limit of TSH reference intervals was 7.80 μU/mL and 474 samples with TSH levels between 7.80 and 20 μU/mL were obtained. There was a possibility that a TSH value within this interval may indicate a positive case of congenital hypothyroidism. Studies conducted in Greece, Italy, Brazil, the United Kingdom, and Northern Ireland have shown that among individuals with confirmed congenital hypothyroidism, using a threshold of TSH greater than 5–6 μU/mL, 60% had a TSH value exceeding 17 μU/mL, while 40% had values ranging between 6 and 17 μU/mL [49]. In the experience of several newborn screening programs in Europe, when the TSH cut-off was lowered from 20 to 10 μU/mL blood, the incidence of congenital hypothyroidism detected increased from 3.7 to 8.6 per 10,000 [50] and 3.8 to 8.7 per 10,000 [41]. Our research only determined TSH reference intervals in healthy neonates with TSH levels less than 20 μU/mL. A limitation of this study was that we did not evaluate serum FT4 and TSH on neonates with TSH values between 7.80 and 20 μU/mL. Further research is necessary to evaluate the TSH cut-off in conjunction with FT4 levels to confirm hypothyroidism. The objective is to potentially lower the TSH threshold below 20 μU/mL, thereby improving the identification of false negatives in newborn screening and facilitating prompt treatment.

## 5. Conclusions

This study offers comprehensive neonatal TSH interval reference data, considering factors such as sex, gestational age, neonatal age at sampling, and parental ethnicity. This information is intended to apply to the broader neonatal population in Indonesia.

## Figures and Tables

**Table 1:** The neonatal TSH interval references are divided by neonatal age in sex, gestational age, and parental ethnicity group after outlier rejection.

	Neonatal Age (H)	TSH (μU/mL)				*p*-Value
		*n*	Median	2.5th Percentile (90%CI)	97.5th Percentile (90% CI)	(Median ^a,b^, 2.5th and 97.5th Percentile ^c^
Male	≤24	3976	2.70	0.90	7.30	<0.05
				(0.90–0.90)	(6.90–7.50)	
	>24–48	11,874	2.00	0.80	6.20	
				(0.80–0.80)	(6.04–6.30)	
	>48–72	8385	1.40	0.73	5.07	
				(0.70–0.78)	(4.98–5.20)	
	>72	8738	1.31	0.70	4.20	
				(0.70–0.70)	(4.05–4.31)	
Female	≤24	3721	2.40	0.87	7.10	
				(0.80–0.90)	(6.70–7.30)	
	>24–48	10,311	1.80	0.80	5.90	
				(0.80–0.80)	(5.80–6.10)	
	>48–72	7377	1.31	0.75	4.80	
				(0.70–0.80)	(4.60–4.90)	
	>72	7991	1.31	0.70	3.50	
				(0.70–0.70)	(3.40–3.60)	
Early-Term	≤24	3022	2.60	0.90	7.04	
				(0.85–0.90)	(6.70–7.30)	
	>24–48	8731	1.90	0.80	6.10	
				(0.80–0.80)	(5.90–6.20)	
	>48–72	5535	1.40	0.73	5.00	
				(0.70–0.80)	(4.70–5.10)	
	>72	5214	1.31	0.70	4.50	
				(0.70–0.70)	(4.30–4.71)	
Full-Term	≤24	3970	2.50	0.88	7.30	
				(0.80–0.90)	(7.00–7.50)	
	>24–48	11,405	1.90	0.80	6.10	
				(0.80–0.80)	(5.90–6.20)	
	>48–72	9056	1.40	0.73	4.90	
				(0.70–0.77)	(4.80–5.00)	
	>72	10,644	1.31	0.70	4.04	
				(0.70–0.70)	(3.90–4.15)	
Late and Post-Term	≤24	708	2.50	0.90	7.58	
				(0.85–0.90)	(6.50–8.10)	
	>24–48	2049	2.00	0.80	6.07	
				(0.80–0.80)	(5.80–6.70)	
	>48–72	1170	1.50	0.80	5.10	
				(0.71–0.80)	(4.80–5.50)	
	>72	1017	1.31	0.70	4.50	
				(0.70–0.72)	(4.20–4.99)	
Javanese–Javanese	≤24	6698	2.50	0.90	7.10	
				(0.85–0.90)	(6.80–7.30)	
	>24–48	19,266	1.90	0.80	6.00	
				(0.80–0.80)	(5.90–6.10)	
	>48–72	12,692	1.40	0.75	4.90	
				(0.71–0.78)	(4.70–5.00)	
	>72	13,327	1.31	0.72	3.45	
				(0.72–0.73)	(3.40–3.50)	
Madurese–Madurese	≤24	880	2.60	0.90	7.80	
				(0.88–0.93)	(7.30–8.5)	
	>24–48	2480	2.00	0.80	7.00	
				(0.80–0.80)	(6.70–7.50)	
	>48–72	2714	1.40	0.72	5.31	
				(0.70–0.80)	(5.00–5.90)	
	>72	2718	1.31	0.70	5.30	
				(0.70–0.70)	(4.90–5.80)	
Javanese–Others	>24–48	225	1.90	0.80	5.97	
				(0.70–0.87)	(5.20–8.30)	
	>48–72	126	1.45	0.77	4.62	
				(0.70–0.85)	(4.10–7.10)	
	>72	192	1.30	0.70	4.80	
				(0.67–0.72)	(4.03–9.04)	
Javanese–Madurese	>48–72	145	1.50	0.70	4.57	
				(0.66–0.80)	(3.87–7.80)	
	>72	218	1.31	0.70	6.28	
				(0.68–0.71)	(5.01–9.60)	

^a^ Kruskal–Wallis H test, ^b^ Mann–Whitney U test, ^c^ Quantile Regression test.

## Data Availability

The data presented in this study are available on request from the corresponding author. The data are not publicly available due to privacy or ethical restrictions.

## Abbreviations

The following abbreviations are used in this manuscript:

TSH	Thyroid-Stimulating Hormone
CLSI	Clinical Laboratory Standards Institute
NSQAP	Newborn Screening Quality Assurance Program
GSP	Genetic Screening Processor
CDC	Centers for Disease Control and Prevention
SPSS	Statistical Package for the Social Sciences
HPT	Hypothalamic–Pituitary–Thyroid
TRH	Thyrotropin Releasing Hormone
